# Genotype – environment correlations in corals from the Great Barrier Reef

**DOI:** 10.1186/1471-2156-14-9

**Published:** 2013-02-22

**Authors:** Petra Lundgren, Juan C Vera, Lesa Peplow, Stephanie Manel, Madeleine JH van Oppen

**Affiliations:** 1 Australian Institute of Marine Science, PMB No 3, Townsville MC, QLD, 4810, Australia; 2 Department of Biology, Penn State University, 208 Mueller Laboratory, University Park, PA, 16802, USA; 3 IRD, UMR 151, Laboratoire Population Environnement et Développement (LPED), Université Aix-Marseille, Marseille, 13331, France

**Keywords:** SNP, *Pocillopora*, *Acropora*, Coral, Transcriptome, Genotype-environment correlation

## Abstract

**Background:**

Knowledge of genetic markers that are correlated to stress tolerance may improve spatial mapping of reef vulnerability and can inform restoration efforts, including the choice of genotypes for breeding and reseeding. In this manuscript we present two methods for screening transcriptome data for candidate genetic markers in two reef building corals, *Acropora millepora* and *Pocillopora damicornis* (types α and β). In *A. millepora,* Single Nucleotide Polymorphisms (SNPs) were pre-selected by targeting genes believed to be involved in the coral thermal stress responses. In *P. damicornis* (type α and β), SNPs showing varying allele frequencies between two populations from distinct environments were pre-selected. Allele frequencies at nine, five and eight of the pre-selected SNP loci were correlated against gradients of water clarity and temperature in a large number of populations along the Great Barrier Reef.

**Results:**

A significant correlation between environmental category and SNP allele frequency was detected in up to 55% of the tested loci, which is an exceptional success rate for these types of tests. In *P. damicornis*, SNP allele frequencies of *β-hexosaminidase* and *Elongation factor 1-α* were significantly correlated to temperature in type α and to temperature and/or water clarity respectively in type β. Type α also showed a correlation between water clarity and SNP allele frequency in a gene of unknown function. In *A. millepora*, allele frequencies at five (*β-gamma crystallin, Galaxin*, *Ubiquitin, Ligand of Numb X2* and *Thioredoxin*) SNP loci showed significant correlations.

**Conclusions:**

After validation of these candidate loci through laboratory or field assessment of relative stress tolerance of colonies harbouring different alleles, it is anticipated that a proportion of these markers may represent the first coral candidate Quantitative Trait Loci for environmental stress tolerance and provide an important genetic tool that can be incorporated into spatial management decisions and restoration efforts of coral reefs. One pertinent example would be to combine spatial data of tolerant populations with genetic connectivity and thus identify high priority conservation reefs and implement targeted coral husbandry and active restoration efforts that use locally- and stress-adapted genotypes.

## Background

Current models of the Earth’s climate over the coming decades predict rapid changes in a range of parameters including temperature, rainfall, and ocean pH [1]. Given the known and forecasted impacts of climate change on coral reef ecosystems [2-6], a priority area in conservation initiatives should be the determination of the amount of adaptive genetic variation, i.e. variation at loci that have an effect on fitness [7], in key habit forming organisms such as corals. Particularly, information on genetic markers that are correlated to stress tolerance will contribute to the accuracy of spatial mapping of reef vulnerability and can be incorporated into controlled restoration efforts.

There are numerous approaches to detect adaptive genetic variation and signatures of local adaptation (reviewed in [8]). In non-model species, population genomics has become the most commonly used approach for identifying genetic loci that are influenced by selection. Population genomics is a genome-wide application of population genetics, whereby a large number of loci are analysed in multiple populations, allowing for the discrimination between locus-specific (selection) and genome-wide (drift and migration) effects [9,10] or for site specific adaptation in panmictic species [11]. Some of the more commonly reported methods include the detection of non-random population divergence at specific loci (F_ST_ outliers) [12-14], elucidating the genetic architecture of specific phenotypes [15], and associating the frequency distribution of alleles to one or more ecological variables (genetic-environment associations, GEAs) [16,17]. To date, evidence for genetic variation that is significantly associated to environmental heterogeneity, fitness and phenotype remains scarce [18-20].

Adaptive genetic variation and its link to phenotypic traits has been widely studied in forestry (reviewed in [21]. However, in many non-model organisms, the discovery of phenotype-linked genetic markers has often been serendipitous. For instance, a correlation between allozyme variation and variable thermal stress tolerance occurs in the fish *Fundulus heteroclitus*, where the gene *Lactate dehydrogenase-B* shows differences in allele frequencies between populations from distinct thermal environments [22]. This variation is responsible for differences in physiological function and survival at high temperatures. Other examples of genotype to phenotype associations include the link between polymorphism in the *Dopamine receptor D4* and personality in great tits (*Parus major*) [23], and the correlation between melanism and variation in the *Melancortin-1 receptor gene (Mc1r)* across several species, including moth, mice and humans [18]. In the panmictic American eel, correlations between environmental variables and SNP allele frequencies have been attributed to post dispersal localised selection [11]. The discovery of links such as those described above remains a challenge. However, the increased accessibility of genomic tools and data from non-model organisms make this a rapidly expanding research area.

The Great Barrier Reef (GBR) is the largest coral reef ecosystem in the world. It spans over 2300 km along the East Coast of Australia and encompasses almost 3000 reefs [24]. Like most of the world’s coral reefs, the GBR is under the influence of a range of direct and indirect anthropogenic stressors. Corals derive a large proportion of their energetic needs from the photosynthetic products of an endosymbiotic dinoflagellate (genus *Symbiodinium*) [25] and thrive in clear, nutrient poor tropical waters, often near their upper thermal tolerance. Not surprisingly, current research shows that two of the main threats to the long term health of the GBR are climate change [5,26] and declining water quality (in particular, water clarity) due to coastal development and catchment runoff [27,28].

The most commonly reported thermal stress response in corals is coral bleaching, which is the dissociation between the *Symbiodinium* and the coral host tissue [2,29-34]. Variation in bleaching resistance within and among coral species can occur as a result of many factors associated with both the coral host and its *Symbiodinium*. In the coral *Acropora millepora,* thermal stress tolerance has been shown to be correlated with ambient water temperatures in three thermally distinct populations [35]. While this is at least partly due to the *Symbiodinium* type harboured [35], patterns of genetic differentiation at coral host allozyme loci are correlated with latitudinal gradients of sea water temperature [36], suggesting past adaptation of the host genome to local environments. Similarly, in the coral *Pocillopora damicornis,* laboratory studies have shown a latitudinal difference in bleaching sensitivity that was not correlated to the type of *Symbiodinium* harboured, indicative of localised adaptation of the coral host to varying thermal regimes [37].

The first studies of gene expression in corals emerged around the turn of the century [38]. Since then, a large number of expressed sequence tags (ESTs) generated through the reverse transcription of gene transcripts (mRNA) expressed during stress, have been made publically available [39-44]. An overlapping subset of stress response genes have been identified in multiple coral species, which allows the identification of key genes and proteins to target for studies of the coral stress response [45]. *Acropora millepora* has emerged as one of the model species for coral genetics and genomics. The species has been the target of multiple experiments on transcriptome effects of environmental stress [41,43,46-50], thus providing comprehensive sequence information for a range of stress response genes. Also, an increasing amount of coral genome and transcriptome data is being made available in public databases [51,52], including the recent release of the first complete coral genome sequences for *Acropora digitifera*[53] and *A. millepora* (http://coralbase.org/). However, the concept of identifying loci, which can be used as markers for relative stress tolerance, remains untested in corals.

This paper presents the first putative genetic markers for environmental stress tolerance in corals. The genetic markers were identified using two novel transcriptome-based strategies. Both strategies make use of next generation sequencing and large scale Single Nucleotide Polymorphism (SNP) genotyping across environmental gradients and build on the concept of identifying GEAs [16]. These strategies increase the likelihood of finding GEAs compared to traditional methods through targeted, population level approaches of transcriptome sequencing and genotyping.

## Methods

Two strategies were followed to identify SNPs that show a significant correlation with environmental gradients, which differ in their use of newly generated versus already available transcriptome data. The strategies are not compared with each other for efficiency, rather presented as two alternative approaches to generate a similar outcome.

The first strategy involved three steps: (1) generate population-tagged transcriptome data from a small number of different environmental zones; (2) mine transcriptome data for SNPs that show a distinct difference in allele frequency between the targeted populations; (3) test the selected SNPs on population samples from a more extensive range of the environmental variable. In the second strategy, step (1) was excluded and step (2) was modified to mining already published transcriptome data for SNPs in pre-selected genes; step (3) was the same in both strategies. Both strategies generated genotypic data across a large number of populations from which allele frequencies were calculated. Correlative methods were used to detect significant genome environment associations (GEAs) as per below.

### A targeted Next Generation sequencing effort in *Pocillopora damicornis*

**Figure 1:**
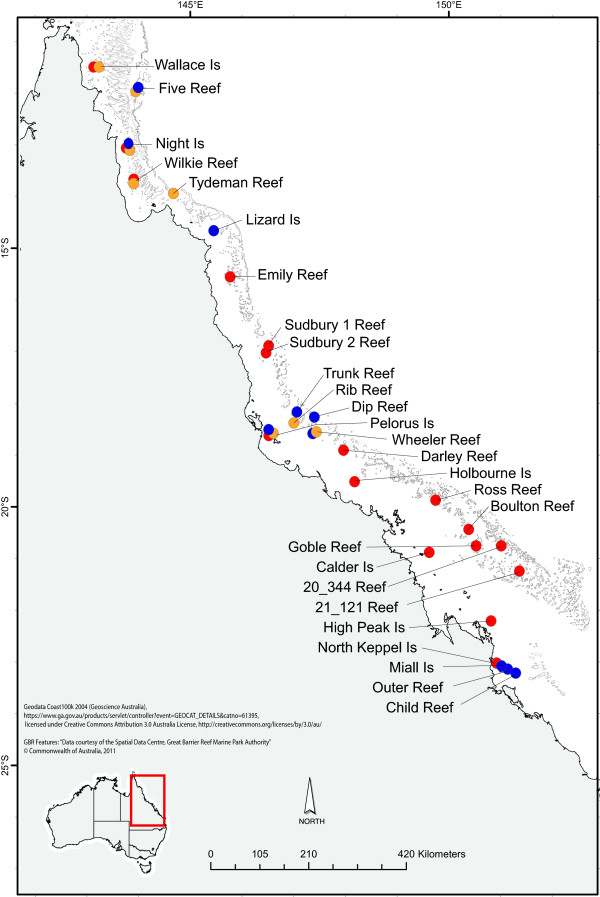
**Map of sampling locations for *P. damicornis *(type α) (blue), *P. damicornis *(type β) (yellow) and *A. millepora *(red).**

**Figure 2:**
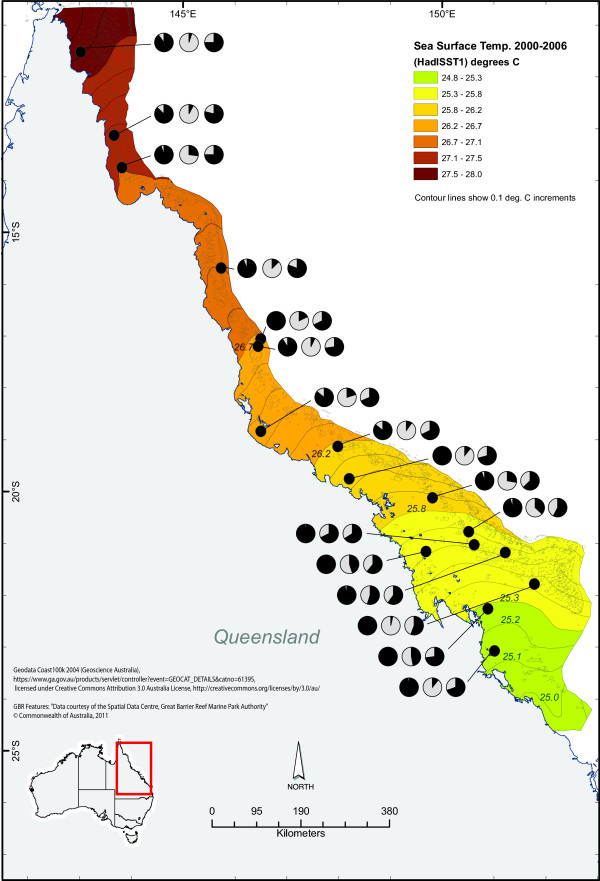
**Map showing allele frequency patterns of three SNP loci (*β-gamma crystallin *right, *Galaxin *middle and *Ubiquitin *left) in *A. millepora *with a significant correlation to temperature categories, plotted on spatial data for average sea surface temperatures between the years 2000 – 2006.** Map courtesy of the e-Atlas (http://e-atlas.org.au).

**Figure 3:**
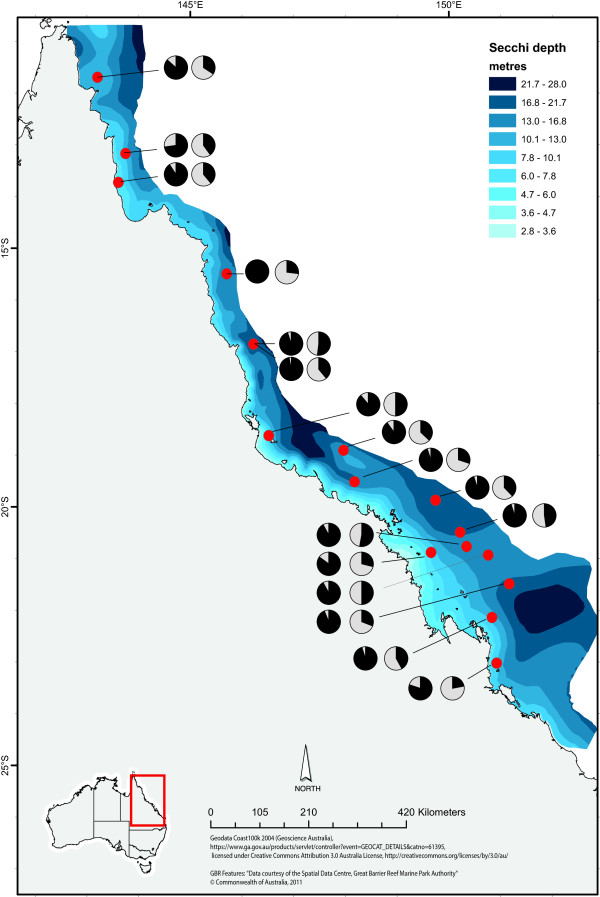
**Map showing allele frequency patterns of two SNP loci (*Thioredoxin *right, *Ligand of numb X2 *left) in *A. millepora *with a significant correlation to water clarity, plotted on spatial data for secchi depths collected between 1992 and 2006.** Map courtesy of the e-Atlas (http://e-atlas.org.au).

Two nubbins from each of 20 colonies of the common reef coral *Pocillopora damicornis* were collected from Miall Island in the southern GBR (Keppel Islands), Pelorus Island in the central GBR (Palm Islands), and two sites in the far northern GBR, Wallace Islet and Wilke Reef (Figure 1), spanning a large gradient of temperature and water clarity (Figures 2 and 3), thereby targeting natural gradients of two major stressors on the Great Barrier Reef (GBR), namely elevated sea surface temperatures and declining water quality [4,54,55]. The nubbins were snap frozen in liquid nitrogen immediately after collection and stored at −80°C until RNA extractions took place.

Recent research has shown that *P. damicornis* comprises several mtDNA lineages [56,57], and on the GBR two major distinct lineages (type α & β) have been identified [58]. Therefore, all samples were genotyped using genetic markers that have been developed to separate these two cryptic, sympatric lineages (Torda et al. un-published material). The results revealed that a majority of the colonies that were collected from Wallace Islet and Miall Island reefs were type α, while the Wilke and Pelorus Island reef samples comprised more colonies of type β. As a result, the collections were divided according to their specific lineage.

Messenger RNA was isolated using a modified protocol of the Invitrogen™ Dynabeads® Oligo(dT)_25_[43]. Two extractions were made from each colony, with a yield of approximately 20 ng/μL per extraction. RNA quality and quantity were verified using the mRNA Pico series kit on the Agilent© 2100 Bioanalyzer. To ensure good quality transcriptome sequences, only samples with low rRNA contamination (<10%) and good quality mRNA were included in the study. As a result, five and six samples of *P. damicornis* (type β) from Wilke and Pelorus Island reefs were used, respectively. These samples were pooled into two population specific samples. For *P. damicornis* (type α), mRNA from three Wallace Islet and two Miall Island samples were pooled into a third sample and included in the 454 analysis. The final sequencing was done for three pooled mRNA samples using the 454® Genome Sequencer FLX Titanium Series reagents according to the manufacturer’s instructions (Roche Diagnostics Corporation) by the Australian Genome Research Facility (AGRF).

The 947,623 resulting sequences and quality scores were analysed using PipeMeta, a menu based, desktop interface pipeline toolset for Windows [59,60]. Sequences in Fasta format were screened for SMART primer (http://www.clontech.com/) using the SmartScreener script from within PipeMeta and then reads and quality scores were trimmed and quality filtered (min/max length: Avg. Length ± 2*SD; minimum avg. quality score: 20; maximum number of degenerate bases: 1). The remaining 653,814 reads and quality scores (73.6% of the original sequencing effort) were then tagged (i.e. α-numeric strings added to titles) by population to differentiate them after assembly. The sample containing the partial transcriptome of *P. damicornis* (type α) was not included in the final assembly, which was done using the sequences from *P. damicornis* (type β) only*.*

Assembly of the timed reads and quality scores (fasta files) was performed using commercially available assembly software (SeqMan NGen 2.1.0 build 13, DNAStar). To further assess the performance of the assembly, all resulting unigenes (Contigs and Singletons over 50 bp) were Blast aligned to a modified set of *in silico* predicted proteins from the starlet sea anemone *Nematostella vectensis*[61] and *Acropora digitifera*[53], the two closest related species to *P. damicornis* (type β) with sequenced and publically available genomes. In order to better estimate transcriptome coverage of the sequencing effort, both sets of predicted genes were modified to remove shorter protein sequences with greater than 90% similarity to longer sequences in the set.

All *P. damicornis* (type β) unigenes were Blast aligned to the Uniprot protein database (http://www.expasy.uniprot.org/database/download.shtml) to annotate the assembly.

### Targeting SNPs in *Pocillopora damicornis* and *Acropora millepora*

The *P. damicornis* (type β) sequence data were searched for functional SNPs using the SNP hunter extension of the PipeMeta software (http://www.personal.psu.edu/jhm10/Vera/software.html), a tool specifically designed to find SNPs in *de novo* transcriptome assemblies using a variety of customizable settings and filters. About 24,000 SNP candidates were located within the partial *de novo* transcriptome assembly and screened to identify potential biomarkers.

The candidate SNPS were narrowed down by applying the following filters. First, all non-annotated contigs were removed, followed by any contigs that did not align to ESTs or genes of known function that were derived from other metazoans (to avoid *Symbiodinium* sequences). Second, all SNPs that did not have a frequency of > 10% in at least one of the two populations were removed to minimize false positives due to sequencing errors. After this, a search was made for bi-allelic SNPs for which allele frequencies differed by > 20% between the two populations (e.g., if one allele occurred at a frequency of 40% in one population, it had to have a frequency of < 20% or >60% in the other to qualify). Based on these criteria, a final list of 80 SNPs distributed among 67 contigs was created.

Primers for the 80 SNPs were designed using Primer 3 (http://frodo.wi.mit.edu/primer3/) to amplify a 75 – 150 base pair PCR product. PCR amplification was performed using DNA extracts from three colony samples of *P. damicornis* (type β) and the product was visualized on a 1% TAE-agarose gel. Any primer pair that produced products that were longer than predicted and hence possibly included intron sequences, yielded inconsistent amplification success or displayed any other signs of poor quality, were discarded. A final selection of eight SNPs was made to use on the larger, GBR wide sample set of *P. damicornis* (type β) (Table 1). Reciprocal BLAST alignment contig IDs from the Taylor-Knowles et al. [62] transcriptome were added to allow comparisons. Five of these were successfully amplified also in *P. damicornis* (type α).

**Table 1:** **List of SNPs and contig information for the eight SNP loci that were included in the final analysis in *P. damicornis *(type β)**

**Gene**	**SNP name**	**Codon pos**	**Putative role in stress response**	**Reciprocal contig from Taylor-Knowles et al.[**[62]**]**
Beta-hexosaminidase *	171_CT_243	3	Immunity response	bu_91849_c19034
Elongation factor 1-alpha (1841) *	1841_GA_723	3	Stress induced apoptosis	bu_91849_c14445
Elongation factor 1-alpha (2631) *	2631_GA_1228	1	Stress induced apoptosis	bu_91849_lrc55786
Putative un-characterised protein 1.1*	24_CT_847	Un-known		bu_91849_c40532
*Putative un-characterised protein1.2*	24_CA_2390	Un-known		bu_91849_c40532
*Putative un-characterized protein – mitochondrial*	269_GA_818	Un-known		bu_91849_c2047
*Carbonic anhydrase*	1361-TC_426	3	A catalyst for the conversion of carbon dioxide and water into bicarbonate and protons	bu_91849_c8064
40S ribosomal protein S3 *	5662_AG_462	3		bu_91849_lrc55459

SNP name constructed in the following manner: *(Contig number_SNP nucleotides_position of SNP in trimmed contig).* Genes marked with * were amplified also for type α. Reciprocal BLASTs were made to match the contigs containing the SNPs with that of the Taylor-Knowles et al. [62] assembly.

Due to the prior knowledge and sequence data for a range of stress response genes in *A. millepora*, the already published transcriptome data set [51] from larvae collected at Magnetic Island, an small inshore island in the central GBR, was downloaded and mined for SNPs using PipeMeta as above. A selection of nine genes was made, based on published information relating to thermal stress responses and SNP information derived from the transcriptome sequence data [51] (Table 2). For each gene, one suitable, non-synonymous SNP site was chosen to be used on the GBR wide sample set (Table 2).

**Table 2:** **Selected genes in *Acropora millepora, *SNP codon position, and their putative role in the stress response**

**Gene**	SNP name*	**SNP codon pos**	**Putative role in stress response**
*Argenine kinase*	50281_AG_478	1	Detoxification of hyper-oxide
*β-gamma crystallin*	35180_TG_523	2	Immunity response
*Coatomer*	60613_TG_230	2	Intra cellular protein transport
*Complete component C3*	26792_AG_293	1	Immunity response
*Galaxin*	20421_TC_239	1	Calcification and growth
*Hsp60*	52394_AG_280	2	Generic heat shock protein
*Mn Superoxide dismutase*	16774_AC_791	1	Defense against ROS
*Thioredoxin*	16728_TC_211	1	Defense against ROS
*Ubiquitin like protein*	12368_TC_731	2	Defense against ROS
*Ligand of numb X2*	63538_CG_709	1	Possible ligase to bind ubiquitin

*contig numbers from Meyer et al. 2009a.

SNP name constructed in the following manner: *(Contig number_SNP nucleotides_position of SNP in trimmed contig).*

### Genotyping along environmental gradients in *Pocillopora damicornis* and *Acropora millepora*

As climate change and water quality are considered among the largest threats to coral reef health on the Great Barrier Reef [5,55], temperature and water clarity were chosen as two highly relevant parameters for stress tolerance. These two environmental variables were extrapolated across the GBR using the e-Atlas tool. Temperature data were derived from Rayner et al. [63] and can be found at http://e-atlas.org.au/content/sea-surface-temperature-hadisst-11 (Figure 2). Secchi disc depth data, which is the most widely available measure of water quality [64], were provided by scientists at the Australian Institute of Marine Science and Queensland Department of Primary Industries and Fisheries and was sourced from http://e-atlas.org.au/content/secchi-disk-depth-measure-water-clarity (Figure 3).

These datasets divide the environmental range into seven temperature and nine secchi disc depth categories, where seven is the highest temperature and nine is the deepest secchi disc depth. All temperature zones were included in the collections. Due to the photosynthetic properties of the coral-*Symbiodinium* symbiosis, corals are rare in highly turbid waters, hence coral collections spanned from secchi disc depth category 4 (6 – 7.8 m) to category 9 (21.7 – 28 m).

Existing samples of *P. damicornis* and *A. millepora* collected for GBR connectivity studies [65] were used for this study. Due to the current putative species delineation of *P. damicornis*[58] all samples of that species had to be identified using the lineage specific genetic markers mentioned above (Torda et al. submitted manuscript).

After species validations, nine populations of *P. damicornis* (type β) were chosen based on having a sample size of ≥ 15 colonies. The larger sample collection of *P. damicornis* (type α) allowed a more comprehensive selection of populations to be made for this species. Ten populations with a sample size of ≥20 colonies, spanning a broader range of thermal categories and at least two replicates of each water clarity category, were chosen for the environmental correlation analyses (Figure 1, Table 3). Eight and five SNP loci were tested in *P. damicornis* (type β) and (type α), respectively, against the two environmental categories, making a total of 26 tests. For *A. millepora*, 9 SNP loci were tested against the same environmental categories (total of 20 tests) across 17 populations, all with sample sizes of ≥ 20 (Figure 1, Table 3) .

**Table 3:** List of sampling sites; location (far north (FN), north, central and south Great Barrier Reef) , reef types (Inshore, Mid shelf (Mid) and Outer), water clarity and thermal categories (each divided into 9 and 7 categories respectively, thermal 1–7 coldest to warmest, water clarity; 1–9 lowest to highest secchi depth) and number of samples of each species

**Reef / Island name**	**Location**	**Reef type**	**Water clarity category**	**Thermal category**	**No samples**		
					***P. damicornis (type β)***	***P. damicornis (type α)***	***A. millepora***
Wallace Isl	FN	Inshore	6	7	15		20
Five Rf	FN	Outer	8	6	15	20	
Night Isl	FN	Inshore	5	6	15	20	20
Wilkie Rf	FN	Inshore	4	6	15		20
Tydeman Rf	FN	Outer	6	6	15		
Lizard Isl	North	Mid	6	5		20	
Emily Rf	North	Mid	7	5			18
Sudbury 1	North	Mid	8	5			20
Sudbury 2	North	Mid	8	5			28
Trunk Rf	Central	Mid	8	4		20	
Rib Rf	Central	Mid	8	4	15		
Dip Rf	Central	Outer	9	4		20	
Pelorus Isl	Central	Inshore	5	4	15	20	20
Wheeler Rf	Central	Mid	9	3	15	20	
Darley Rf	Central	Mid	7	3			20
Holbourne Isl	Central	Inshore	7	3			20
Ross Rf	Central	Mid	8	3			20
Boulton Rf	Central	Mid	8	2			37
Goble Rf	South	Mid	7	2			20
Calder Isl	South	Mid	5	2			20
20_344	South	Mid	7	2			18
21_121	South	Mid	8	2			19
High peak Isl	South	Inshore	6	1			20
North Keppel Isl	South	Inshore	5	1			28
Miall Isl	South	Inshore	5	1		20	
Outer Rf	South	Inshore	6	1		20	
Child Rf	South	Inshore	6	1		20	

DNA was extracted from a small fragment of coral, following a slightly modified method by Wilson et al. [66]. For both *P. damicornis* types the SNP genotyping step was done using qPCR High Resolution Melt curve analyses (HRM) (QIAcube ©, QIAGEN) in the following manner: The PCR step was set up in 10 μL reactions incorporating Accumelt HRM supermix (Quanta Biosciences©). Each sample was set up in triplicate with a primer concentration of between 200 nM and 500 nM, and 1 μl of 1/50 diluted gDNA. No template (negative) and known genotype (positive) controls were included in all runs. PCR and HRM were carried out under the conditions recommended by the instrument manufacturer. PCR commenced with an initial 5 minute hold at 95°C followed by 40 cycles of a 10 second, 95°C denaturation and 30 second, 55°C annealing and extension. For the HRM analysis the temperature was ramped from 65°-95°C, rising by 0.1°C each step with a 90 second pre-melt conditioning cycle on the first step and a 2 second wait every cycle thereafter. Prior to HRM, the gain was optimised to set the greatest fluorescence to less than 90 fluorescence units.

Control samples of identified genotypes were included in each PCR /HRM run. CT values and amplification completeness were checked using the Rotorgene Q series software V2.02 and samples showing late or poor amplification were excluded. HRM profiles were normalised against average signatures within a pre-selected temperature range. The control samples were used as references to automatically determine sample genotypes. All genotype calls were checked manually, and confirmed by further analysis using ScreenClust (Qiagen) software.

For *A. millepora*, genotyping was conducted at the AGRF using a Sequenom® MassArray on an Autoflex Spectrometer using iPLEX GOLD chemistry.

### Statistical analyses

Correlations between environmental variables and SNP allele frequency were tested by using a logistic regression with a logit link and a binomial error distribution to relate the probability of allele 1 in each locus to the two environmental variables, temperature and secchi depth [67]. The likelihood ratio and Wald tests were used to determine the significance of the models as in Joost et al. [68].

To test for the proportion of random correlations, 159 alleles from 11 putatively neutral microsatellite loci were also tested for these environment correlations using the already published *A. millepora* microsatellite data set from the same coral samples [65].

## Results

### Pocillopora damicornis

#### Transcriptome sequencing

After preliminary machine quality filtering, 947,623 sequences were obtained. Additional primer screening and quality filtering through PipeMeta removed 27.4% of all sequenced nucleotides. The remaining 653,814 reads were used in the assembly. Roughly 67% of the trimmed, filtered reads (64% of the sequenced nucleotides) assembled to form 87,520 contigs with an average length of 584 (±296) base pairs (bp), with the 244,541 remaining singletons averaging 304 (±85) bp in length (Table 4).

**Table 4:** **Summary of 454 sequence data for *P. damicornis *(type β)**

Number of nucleotides	277 428 160
Number of sequences	947 623
Average (trimmed) sequence length	312.5 (±85 sd)
Number of assembled contigs	87 520
Average length of contigs	584 (±296)
Proportion significant BLASTs – percent of annotated contigs	20%
% host gene (of total/of annotated data)	13.6% / 68%

To further assess the quality and coverage achieved by the assembly, the unigenes were BLAST aligned to two reference gene sets from closely related species with sequenced genomes (i.e. *Nematostella vectensis* and *Acropora digitifera* modified *in silico* predicted proteins). Out of these, 10,021 (23.4%) and 11,148 (52.4%) were unique hits to *N. vectensis* and *A. digitifera*, respectively. Using a conservative approach to estimate transcriptome coverage length, approximately 16.0% of *N. vectensis* total amino acids and 25.3% of the *A. digitifera* total amino acids were covered (i.e. had strong alignment) by *P. damicornis* (type β) unigene sequence, with median coverage rates of 35.6% and 38.7% per predicted gene with a strong alignment for *N. vectensis* and *A. digitifera* proteins. Less than 6.8% (*N. vectensis*) or 10.2% (*A. digitifera*) of the above reference gene amino acid coverage was from more than one *P. damicornis* (type β) unigene (i.e. duplicate or overlapping amino acid coverage by multiple unigenes).

A BLAST alignment versus the Uniprot protein database produced significant hits (e-value cut off of 1×10^-3^; bitscore > 45) for 54,094 or 61.8% of the Contigs, out of which 44.5% had top alignments to proteins expressed in metazoans , 15.2% aligned to plant, 8.4% aligned to bacteria, and 0.3% aligned to virus (Table 4).

#### SNPs

From the eight SNPs that were selected from the transcriptome of *P. damicornis* (type β) only one was a transversion (24_CA_2390). This SNP occurred at Night and Tydeman reefs with the rare allele (A) occurring at a frequency of 18.1% and 7.6% respectively. In the original transcriptome data, this allele occurred at Wilke Islet reef, which is located in the same geographic region as Night and Tydeman (Figure 1). The remaining seven SNPs were all transitions (C/T or G/A). The resolution of the single step PCR to HRM method used in this study did not allow discrimination of A to T or C to G mutations. Hence, all the SNPs analysed by this method caused a change in the number of hydrogen bonds in the DNA chain, which produced a large enough change in the melting temperature to allow accurate scoring.

The final data set for *P. damicornis* (type β) was based on the allele frequencies at eight SNP loci. In *P. damicornis* (type α)*,* five loci were amplified using the primers developed for *P. damicornis* (type B) (Table 1). The other three loci were excluded from trials in *P. damicornis* (type α) due to ambiguous scoring patterns of the HRM curves in this species (see Additional file 1: Figure S1 for examples of HRM curves). In *P. damicornis,* the SNPs were found in genes of known and unknown function and included both synonymous and non-synonymous mutations. The genes of known function included *Elongation factor 1-α, β-hexosamidase, Carbonic anhydrase* and the *40S ribosomal protein S3* (Table 1).

#### Allele frequency and environment correlations

**Figure 4:**
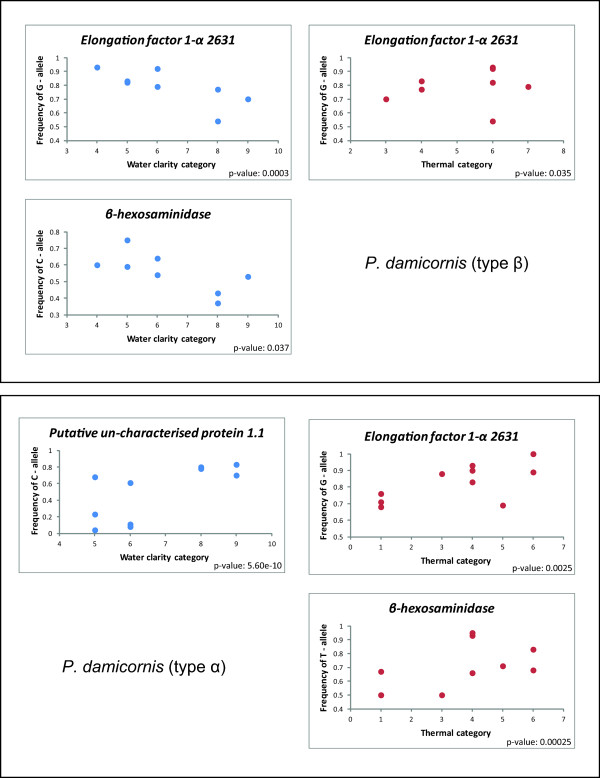
**Scatter plots of SNP allele frequencies and environmental categories in *P. damicornis *(type α) and (type β).**

Using the logistic regression approach, three of the eight tested SNP loci had at least one significant correlation in either *P. damicornis* type α or type β (Table 5; SNP allele frequencies are presented in Additional file 2: Tables S1 and Additional file 3: Table S2). Calculated as number of loci with significant frequency correlations, two out of eight (25%) were significant in *P. damicornis* (type β) and three out of five (60%) were significant in *P. damicornis* (type α). These correlations were found between temperature and SNP allele frequency in *β-hexosamidase* in *P. damicornis* (type α) and for water clarity in *P. damicornis* (type β). Temperature and allele frequency was also significantly correlated to SNP allele frequency in *Elongation factor 1-α_2631* for type α and β as well as a gene of unknown function (*Contig_24_847)* in *P. damicornis* (type β). *P. damicornis* (type β) also showed a correlation between water clarity and SNP allele frequencies in *Elongation factor 1-α_2631* (Table 5; Figure 4).

**Table 5:** **Summary of statistics for the SNP loci that show a significant correlation between allele frequency distribution and environmental category in *P. damicornis *(type α & β)**

**Gene**	**Environmental variable**	**P. damicornis (type α)**		**P. damicornis (type β)**	
		**Coeff LR**	**p-value**	**Coeff LR**	**p-value**
*β-hexosaminidase*	Temp	0.250	**0.00025**	0.121	0.396
	Water clarity	0.114	0.213	0.190	**0.0374**
*Elongation factor 1-α 2631*	Temp	0.232	**0.0025**	0.376	**0.0347**
	Water clarity	0.126	0.246	0.472	**0.0003**
*Putative un-characterised protein 1.1*	Temp	0.057	0.399	0.085	0.538
	Water clarity	0.594	**5.60e-10**	0.539	0.051

Coeff LR = regression coefficients of the logistic regressions, p-values derived from the Wald tests and considered significant when < 0.05.

### Acropora millepora

**Figure 5:**
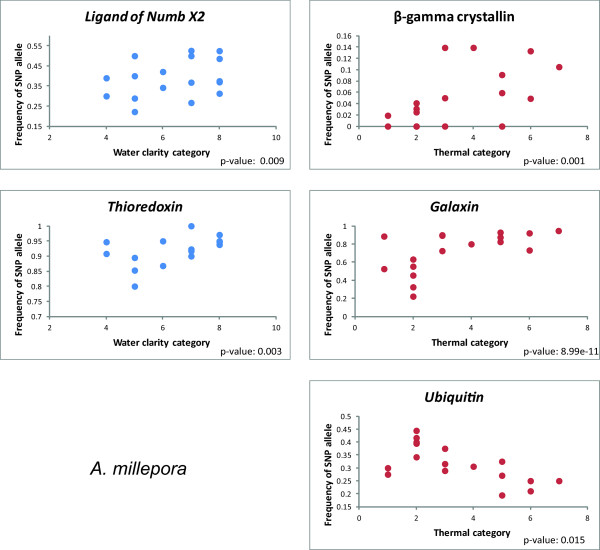
**Scatter plots of SNP allele frequencies and environmental categories in *A. millepora. ***

For the *A. millepora* data set, the Sequinom® MassArray produced sequence data, hence allowing the inclusion of the C to G mutation that was evident in the *Ligand of Numb X2* locus. One of the nine chosen loci (*Coatomer*) was found to be monomorphic and excluded from further analyses. The remaining eight SNPs were successfully genotyped in the majority of the colony samples. Allele frequencies in five (55%) of these loci showed a significant correlation with environmental gradients. *β-gamma crystallin, Galaxin* and *Ubiquitin* were correlated to temperature and *Thioredoxin* and *Ligand of Numb X2* were found to correlate to water clarity (Table 6; Figures 2, 3 and 5).

**Table 6:** **Summary of statistics for the SNP loci that show a significant correlation between allele frequency distribution and environmental category in *A. millepora ***

**Gene**	**Environmental variable**	**Coeff RL**	**p-value**
*Ligand of Numb X2*	Water clarity	0.150	0.009
*Thioredoxin*	Water clarity	0.301	0.003
*β-gamma crystallin*	Temp	0.322	0.001
*Galaxin*	Temp	0.412	8.88e-11
*Ubiquitin*	Temp	0.126	0.0149

Coeff LR = regression coefficients of the logistic regression, p-values derived from the Wald tests and considered significant when < 0.05.

Based on results from the microsatellite data, approximately 10% (16 out of 159 tested alleles, from 9 of the 11 tested loci) were found to have significant correlations purely by chance (Additional file 4: Table S3).

## Discussion

This study presents two novel strategies to identify SNP loci that are correlated to environmental gradients, with the ultimate aim being the discovery of candidates for quantitative trait loci (QTLs) for environmental stress tolerance through subsequent correlative tests of phenotypic response. Both methods revealed likely signatures of selection in up to 55% of the loci tested. This is a considerable improvement compared to the previously reported 2-10% in non-targeted, genome-wide scans for outliers [69].

Of a small subset of SNPs screened across a large spatial scale on the Great Barrier Reef (GBR), four, two and three SNP(s) were found to be correlated to either temperature or water clarity in the reef-building coral species *Acropora millepora, Pocillopora damicornis* (type α) and *Pocillopora damicornis* (type β), respectively. These represent the first genetic markers in corals for environmental stress tolerance. After validation of these candidate loci, i.e., through laboratory or field assessment of relative stress tolerance of colonies harbouring different alleles at these loci, it is anticipated that a proportion of these loci can be used as *de facto* QTLs.

The risk of confounding effects (e.g. population history, neutral “surfing” allele of an expanding population, endogenous genetic barrier) causing false positives as discussed in Bierne et al. [19] is reduced by actively selecting SNPs for either function or population divergence prior to being tested for correlations. Furthermore, test on independent allelic data at 11 putatively neutral microsatellite loci in *A. millepora* provided an estimated the occurrence of false positives to be a maximum of ~10%.

All the SNPs that were selected in *A. millepora* and at least one (*EF-1-α )* of the three significant SNPs in *P. damicornis* were non-synonymous mutations (Tables 1 and 2), hence are likely to be non-neutral makers upon which selection can act, either directly through a change in expression or function, or through linkage to other loci that are under selection. β- gamma-crystallin is mostly studied in relation to cataracts in humans and other model vertebrates [70]. The protein has also been shown to bind calcium in bacteria [71] and cephalochordates [72]. The binding of Ca2+ influences the stability of the protein and thus alters its thermal denaturation points [71], which may correlate to more successful calcification at higher temperatures. In corals, expression levels of *β- gamma-crystallin* has been shown to have a high heritability (h^2^ = 0.38), indicative of the existence of adaptive variation, and have been correlated to variations in larval responses to elevated temperatures and settlement cues [48]. Another gene that is involved in the calcification process, which exhibits allele frequency distributions that correlate to thermal gradients in the present study, is galaxin*.* The protein this genes encodes was originally characterised from the coral *Galaxea fascicularis* and is a soluble protein from the coral exoskeleton, where it is involved in calcification [73]. Three genes that are related to galaxin, amgalaxin , amgalaxin like-1 and amgalaxin like-2 have been characterised in *A. millepora*. The two amgalaxin like proteins are expressed exclusively during the early stages of calcification in the newly settled coral polyp, while amgalxin is continuously expressed also in the adult colony [74]. Recent studies indicate that temperature plays a pivotal role in coral calcification rates [26], supporting the correlation between temperature and proteins involved in the calcification process observed here. Ubiquitin is a protein that targets damaged proteins and marks them for destruction, a process referred to as ubiquitylation [75]. Thus, measurements of relative levels of ubiquitin provide an index of the structural integrity of cellular proteins and have been used as a biomarker for cellular stress in corals [76]. Differences in levels of ubiquitin conjugated proteins remain fixed during reciprocal transplantation of the coral *Porites lobata*, with corals from environmentally variable back reef sites showing consistently higher levels, regardless of transplant location [77], which adds to the current indication of a genetic difference contributing to variations to environmental stress response. SNP allele frequencies in the *Ligand of numb X2* and *Thioredoxin* genes correlated to water clarity. Ligand of numb X2 is a close homologue to the Ligand of numb X, which is an important ligase for ubiquitin [78] while thioredoxin is involved in the sequestration of reactive oxygen species that are produced during oxidative stress [79]. Further to its putative role during oxidative stress, thioredoxin has been shown to be significantly differentially expressed along pollution gradients in the Bermudas [40], indicating that this protein is involved in the response to a range of stressors, including water quality.

In *P. damicornis*, two genes of known function were found to have significant correlations with temperature and/or water clarity, namely *EF 1-α* and *β-hexosaminidase.* Apart from its role in the process of cellular binding of actin [80], *EF 1-α* has been shown to be significantly down-regulated during cancer cell senescence [81] and during cell apoptosis associated with other cell malfunctions such as tetraploidy [82]. Hence, a possible change in function of this protein may impact the onset of apoptosis in stressed corals. β-hexosaminidase is involved in a range of functions including the glycosalation of proteins, cleaving of N-acetylgalactosamine (GalNAc) residues that form part of the cell signaling pathway, and correct protein folding mechanisms. It has also been shown to play a role in mycobacterium defense [83], which suggests it may play a role in the immune response. It is well documented that stressed corals show increased susceptibility to disease [84-86], hence a correlation between an immune response and increasing temperature or declining water quality is expected.

Although phenotypic responses are rarely determined by a single locus, but rather by the cumulative small effects of multiple loci [15], it has been observed in some studies that SNPs may be responsible for a large part of the variance in a specific phenotypic trait [11,23,87]. Hence, the direct utility of DNA-based QTLs is that the presence or absence of a certain allele is indicative of the relative stress tolerance of an individual or a population, as was shown to be the case of the relative proportion of one LDH-β allozyme and its correlation to increased thermal stress tolerance in populations of the killifish *F. heteroclitus*[22]. The use of QTLs is widespread in crop science, where extensive QTL mapping is used to direct controlled breeding for desired traits such as drought and pest resistance [88]. The link between genotype at the SNP loci presented here and the coral phenotype is currently being validated through aquarium experiments and *in situ* observations.

## Conclusion

Developing and publishing efficient methods to screen genomes and transcriptomes for candidate genetic markers for stress tolerance remains an important scientific endeavour, and the development of temperature and water clarity-associated genetic markers in corals is both timely and relevant. Climate change does not only mean increasing sea surface temperatures, it is also predicted to come with an increasing occurrence of extreme weather events [89], including heavy rainfall, which results in increased influx of sediment, agricultural runoff and fresh water into the GBR lagoon, all of which negatively impact near shore coral reefs [90]. In addition, the GBR is currently facing a range of acute water clarity impacts such as dredging for port expansions or underwater mining operations. The value of markers such as the ones developed in this study in management practices lies in the potential to identify key reefs and populations that harbour tolerant genotypes and hence are likely to show greater resilience to increasing sea surface temperatures or declining water quality. A combination of high resilience and a role as a source reef for coral recruits would provide a solid foundation for the implementation of protection of a particular reef [91]. Other potential applications of genetic markers for stress tolerance include the potential to identify more stress tolerant colonies for seeding of degraded reefs, to initiate selective breeding programs, or to actively preserve certain genotypes for future breeding and restoration efforts.

## Authors’ contributions

The manuscript is the outcome of the postdoctoral research of PL, who conceptualised the idea and acquired funding in collaboration with MvO, collected field material, conducted a majority of the pre-transcription laboratory work, was involved in the bioinformatics and subsequent genotyping work and did the majority of the manuscript writing. In addition to the above conceptualisation and fund acquisition, MvO provided core funds and supervision. JCV did the majority of the bioinformatics on the transcriptomes, SM provided insights and support on the statistical analyses, wrote the scripts for the logistic regression analyses and interpreted the results and LP did a large part of the HRM analyses for the *P. damicornis* samples. All authors read and approved the final manuscript.

## Supplementary Material

Additional file 1: Figure S1 Examples of good and ambiguous HRM melt curves. Click here for file

Additional file 2: Table S1 SNP allele frequencies in each population of the two types of *Pocillopora damicornis.* Click here for file

Additional file 3: Table S2 SNP allele frequencies in each population of *Acropora millepora.* Click here for file

Additional file 4: Table S3 Microsatellite alleles from the tested colonies and populations *Acropora millepora* that showed a significant correlation, presumably by chance. Click here for file
