# Supplementary material for: Genotype – environment correlations in corals from the Great Barrier Reef
Source: BMC Genet. 2013 Feb 22;14:9. doi: 10.1186/1471-2156-14-9 (PMC3599201; doi:10.1186/1471-2156-14-9)
Supplement: Additional file 1: Figure S1 — Examples of good and ambiguous HRM melt curves. [file 1471-2156-14-9-S1.docx]

Additional table 1: SNP allele frequencies in each population of the two types of *Pocillopora damicornis*

| Species | *P. damicornis* (type A) | | | | | | | | | | *P. damicornis* (type B) | | | | | | | | | | | | | | | |
| --- | --- | --- | --- | --- | --- | --- | --- | --- | --- | --- | --- | --- | --- | --- | --- | --- | --- | --- | --- | --- | --- | --- | --- | --- | --- | --- |
| Gene name | *Beta-hexosaminidase* | | *Elongation factor 1-alpha (1841)* | | *Elongation factor 1-alpha (2631)* | | *Putative un-characterised protein 1.1* | | *40S ribosomal protein S3* | | *Beta-hexosaminidase* | | *Elongation factor 1-alpha (1841)* | | *Elongation factor 1-alpha (2631)* | | *Putative un-haracterised protein 1.1** | | *Putative un-characterised protein1.2* | | *Putative un-characterised protein – mithochondrial* | | *Carbonic anhydrase* | | *40S ribosomal protein S3* | |
| Population  Allele freq | C | T | G | A | G | A | C | T | A | G | C | T | G | A | G | A | C | T | C | A | C | A | G | A | A | G |
| Wallace Isl |  |  |  |  |  |  |  |  |  |  | 0.54 | 0.46 | 0.90 | 0.1 | 0.79 | 0.21 | 0.50 | 0.50 | 1 | 0 | 0.75 | 0.25 | 0.89 | 0.11 | 0.64 | 0.36 |
| Five Rf | 0.32 | 0.68 | 0.81 | 0.19 | 0.89 | 0.11 | 0.80 | 0.20 | 0.92 | 0.08 | 0.43 | 0.57 | 1 | 0 | 0.54 | 0.46 | 0.57 | 0.43 | 1 | 0 | 0.90 | 0.10 | 0.67 | 0.33 | 0.67 | 0.33 |
| Night Isl | 0.17 | 0.83 | 1 | 0 | 1 | 0 | 0.04 | 0.96 | 0.68 | 0.32 | 0.59 | 0.41 | 0.82 | 0.18 | 0.82 | 0.18 | 0.59 | 0.41 | 0.82 | 0.18 | 0.70 | 0.30 | 0.46 | 0.54 | 0.65 | 0.35 |
| Wilkie Rf |  |  |  |  |  |  |  |  |  |  | 0.60 | 0.40 | 0.93 | 0.07 | 0.93 | 0.07 | 0.17 | 0.83 | 1 | 0 | 1 | 0 | 0.27 | 0.73 | 0.50 | 0.50 |
| Tydeman Rf |  |  |  |  |  |  |  |  |  |  | 0.64 | 0.36 | 1 | 0 | 0.92 | 0.08 | 0.67 | 0.33 | 0.92 | 0.08 | 0.58 | 0.42 | 1 | 0 | 0.67 | 0.33 |
| Lizard Isl | 0.29 | 0.71 | 1 | 0 | 0.69 | 0.31 | 0.61 | 0.39 | 0.81 | 0.19 |  |  |  |  |  |  |  |  |  |  |  |  |  |  |  |  |
| Trunk Rf | 0.34 | 0.66 | 1 | 0 | 0.93 | 0.08 | 0.78 | 0.22 | 0.82 | 0.18 |  |  |  |  |  |  |  |  |  |  |  |  |  |  |  |  |
| Rib Rf |  |  |  |  |  |  |  |  |  |  | 0.37 | 0.63 | 1 | 0 | 0.77 | 0.23 | 0.64 | 0.36 | 1 | 0 | 0.81 | 0.19 | 0.07 | 0.93 | 0.80 | 0.20 |
| Dip Rf | 0.07 | 0.93 | 0.94 | 0.06 | 0.90 | 0.10 | 0.83 | 0.17 | 0.82 | 0.18 |  |  |  |  |  |  |  |  |  |  |  |  |  |  |  |  |
| Pelorus Isl | 0.05 | 0.95 | 1 | 0 | 0.83 | 0.17 | 0.23 | 0.77 | 0.85 | 0.15 | 0.75 | 0.25 | 1 | 0 | 0.83 | 0.17 | 0.60 | 0.40 | 1 | 0 | 0.57 | 0.43 |  |  | 0.75 | 0.25 |
| Wheeler Rf | 0.50 | 0.50 | 0.90 | 0.10 | 0.88 | 0.12 | 0.70 | 0.30 | 0.86 | 0.14 | 0.53 | 0.47 | 1 | 0 | 0.70 | 0.30 | 0.43 | 0.57 | 1 | 0 | 0.90 | 0.10 | 1 | 0 | 0.68 | 0.32 |
| Miall Isl | 0.50 | 0.50 | 1 | 0 | 0.71 | 0.29 | 0.68 | 0.32 | 0.88 | 0.12 |  |  |  |  |  |  |  |  |  |  |  |  |  |  |  |  |
| Outer Rf | 0.33 | 0.67 | 1 | 0 | 0.76 | 0.24 | 0.11 | 0.89 | 0.68 | 0.32 |  |  |  |  |  |  |  |  |  |  |  |  |  |  |  |  |
| Child Rf | 0.50 | 0.50 | 1 | 0 | 0.68 | 0.32 | 0.08 | 0.92 | 0.85 | 0.15 |  |  |  |  |  |  |  |  |  |  |  |  |  |  |  |  |
