# Supplementary material for: Genotype – environment correlations in corals from the Great Barrier Reef
Source: BMC Genet. 2013 Feb 22;14:9. doi: 10.1186/1471-2156-14-9 (PMC3599201; doi:10.1186/1471-2156-14-9)
Supplement: Additional file 2: Table S1 — SNP allele frequencies in each population of the two types of Pocillopora damicornis. [file 1471-2156-14-9-S2.docx]

Additional table 2: SNP allele frequencies in each population of *Acropora millepora*

| Gene name | *Argenine kinase* | | *Βeta-gamma crystallin* | | *Complete component C3* | | *Galaxin* | | *Hsp60* | | *Mn Superoxide dismutase* | | *Thioredoxin* | | *Ubiquitin like protein* | | *Ligand of numb X2* | |
| --- | --- | --- | --- | --- | --- | --- | --- | --- | --- | --- | --- | --- | --- | --- | --- | --- | --- | --- |
| Population  Allele freq | A | G | T | G | A | G | T | C | A | G | A | C | T | C | T | C | C | G |
| Wallace Isl | 0.60 | 0.40 | 0.90 | 0.11 | 0.35 | 0.65 | 0.05 | 0.95 | 0.05 | 0.95 | 0.44 | 0.56 | 0.87 | 0.13 | 0.75 | 0.25 | 0.34 | 0.66 |
| Night Isl | 0.50 | 0.50 | 0.87 | 0.13 | 0.35 | 0.65 | 0.08 | 0.92 | 0.08 | 0.93 | 0.40 | 0.60 | 0.74 | 0.27 | 0.79 | 0.21 | 0.40 | 0.60 |
| Wilkie Rf | 0.50 | 0.50 | 0.95 | 0.05 | 0.46 | 0.54 | 0.27 | 0.73 | 0.06 | 0.95 | 0.43 | 0.57 | 0.91 | 0.09 | 0.75 | 0.25 | 0.39 | 0.61 |
| Emily Rf | 0.63 | 0.37 | 0.94 | 0.06 | 0.53 | 0.47 | 0.13 | 0.88 | 0.03 | 0.97 | 0.43 | 0.57 | 1.00 | 0.00 | 0.81 | 0.19 | 0.27 | 0.73 |
| Sudbury 1 | 0.58 | 0.42 | 1.00 | 0.00 | 0.45 | 0.55 | 0.18 | 0.83 | 0.08 | 0.93 | 0.58 | 0.43 | 0.95 | 0.05 | 0.68 | 0.33 | 0.53 | 0.48 |
| Sudbury 2 | 0.64 | 0.36 | 0.91 | 0.09 | 0.46 | 0.54 | 0.07 | 0.93 | 0.02 | 0.98 | 0.40 | 0.60 | 0.97 | 0.03 | 0.73 | 0.27 | 0.37 | 0.63 |
| Pelorus Isl | 0.55 | 0.45 | 0.86 | 0.14 | 0.43 | 0.58 | 0.20 | 0.80 | 0.05 | 0.95 | 0.58 | 0.43 | 0.90 | 0.11 | 0.69 | 0.31 | 0.50 | 0.50 |
| Darley Rf | 0.53 | 0.47 | 0.86 | 0.14 | 0.38 | 0.63 | 0.10 | 0.90 | 0.18 | 0.83 | 0.43 | 0.58 | 0.90 | 0.10 | 0.68 | 0.32 | 0.37 | 0.63 |
| Holbourne Isl | 0.48 | 0.53 | 1.00 | 0.00 | 0.42 | 0.58 | 0.11 | 0.90 | 0.08 | 0.93 | 0.45 | 0.55 | 0.95 | 0.05 | 0.71 | 0.29 | 0.30 | 0.70 |
| Ross Rf | 0.40 | 0.60 | 0.95 | 0.05 | 0.53 | 0.47 | 0.28 | 0.73 | 0.05 | 0.95 | 0.47 | 0.53 | 0.95 | 0.05 | 0.63 | 0.38 | 0.38 | 0.63 |
| Boulton Rf | 0.45 | 0.55 | 0.96 | 0.04 | 0.53 | 0.47 | 0.37 | 0.63 | 0.05 | 0.95 | 0.41 | 0.59 | 0.95 | 0.05 | 0.58 | 0.42 | 0.49 | 0.51 |
| Goble Rf | 0.40 | 0.61 | 0.98 | 0.03 | 0.58 | 0.43 | 0.68 | 0.33 | 0.05 | 0.95 | 0.30 | 0.70 | 0.92 | 0.08 | 0.66 | 0.34 | 0.53 | 0.47 |
| Calder Isl | 0.50 | 0.50 | 1.00 | 0.00 | 0.19 | 0.81 | 0.45 | 0.55 | 0.03 | 0.98 | 0.33 | 0.68 | 0.85 | 0.15 | 0.61 | 0.39 | 0.29 | 0.71 |
| 20_344 | 0.17 | 0.83 | 0.97 | 0.03 | 0.60 | 0.40 | 0.55 | 0.46 | 0.00 | 1.00 | 0.39 | 0.61 | 0.92 | 0.08 | 0.60 | 0.40 | 0.50 | 0.50 |
| 21_121 | 0.29 | 0.71 | 1.00 | 0.00 | 0.67 | 0.33 | 0.78 | 0.22 | 0.00 | 1.00 | 0.44 | 0.56 | 0.94 | 0.06 | 0.56 | 0.44 | 0.31 | 0.69 |
| High peak Isl | 0.42 | 0.58 | 1.00 | 0.00 | 0.50 | 0.50 | 0.47 | 0.53 | 0.03 | 0.98 | 0.40 | 0.60 | 0.95 | 0.05 | 0.73 | 0.28 | 0.42 | 0.58 |
| North Keppel Isl | 0.70 | 0.30 | 0.98 | 0.02 | 0.47 | 0.53 | 0.11 | 0.89 | 0.10 | 0.90 | 0.48 | 0.52 | 0.80 | 0.20 | 0.70 | 0.30 | 0.22 | 0.78 |
