# Supplementary material for: Genotype – environment correlations in corals from the Great Barrier Reef
Source: BMC Genet. 2013 Feb 22;14:9. doi: 10.1186/1471-2156-14-9 (PMC3599201; doi:10.1186/1471-2156-14-9)
Supplement: Additional file 3: Table S2 — SNP allele frequencies in each population of Acropora millepora. [file 1471-2156-14-9-S3.docx]

Additional table 3: Microsatellite alleles from the tested colonies and populations *Acropora millepora* that showed a significant correlation, presumably by chance.

| Microsatellite loci | Allele | Coeff LR Turbidity | p-value | Coeff LR Temperature | p-value |
| --- | --- | --- | --- | --- | --- |
| AM2_002 | 96 | 0.387 | 3.16E-05 | -0.023 | 6.71E-07 |
| AM2_010 | 152 | 1.238 | 8.42E-06 | 1.459 | 2.39E-05 |
|  | 154 | 0.556 | 3.54E-16 | 0.617 | 4.89E-18 |
|  | 160 | 0.281 | 0.0006 | 0.453 | 1.15E-07 |
| AM2_022 | 159 | 0.041 | 0.475 | 0.304 | 1.44E-08 |
|  | 161 | 0.445 | 1.82E-13 | -0.491 | 2.50E-15 |
| AM2_023 | 131 | 0.225 | 1.57E-08 | -0.121 | 0.001494 |
|  | 133 | -0.204 | 1.34E-10 | 0.090 | 0.0025 |
| WGS_035 | 174 | 0.057 | 0.115 | 0.132 | 9.25E-05 |
| WGS_134 | 128 | 0.518 | 0.0002 | -0.666 | 1.08E-05 |
| WGS_152 | 105 | -0.115 | 0.0005 | 0.151 | 8.16E-07 |
|  | 113 | 0.521 | 2.45E-15 | -0.448 | 7.83E-12 |
|  | 115 | 0.009 | 0.929 | -0.531 | 2.27E-06 |
| WGS_189 | 212 | -0.067 | 0.668 | -0.683 | 9.65E-05 |
| WGS_196 | 132 | 0.652 | 3.83E-06 | -0.781 | 6.71E-07 |
|  | 216 | 0.289 | 0.120 | 0.908 | 1.03E-05 |
